# Hippocampal Adaptive Response Following Extensive Neuronal Loss in an Inducible Transgenic Mouse Model

**DOI:** 10.1371/journal.pone.0106009

**Published:** 2014-09-03

**Authors:** Kristoffer Myczek, Stephen T. Yeung, Nicholas Castello, David Baglietto-Vargas, Frank M. LaFerla

**Affiliations:** Department of Neurobiology and Behavior and Institute for Memory Impairments and Neurological Disorders, University of California Irvine, Irvine, California, United States of America; Universidad de Sevilla, Spain

## Abstract

Neuronal loss is a common component of a variety of neurodegenerative disorders (including Alzheimer's, Parkinson's, and Huntington's disease) and brain traumas (stroke, epilepsy, and traumatic brain injury). One brain region that commonly exhibits neuronal loss in several neurodegenerative disorders is the hippocampus, an area of the brain critical for the formation and retrieval of memories. Long-lasting and sometimes unrecoverable deficits caused by neuronal loss present a unique challenge for clinicians and for researchers who attempt to model these traumas in animals. Can these deficits be recovered, and if so, is the brain capable of regeneration following neuronal loss? To address this significant question, we utilized the innovative CaM/Tet-DT_A_ mouse model that selectively induces neuronal ablation. We found that we are able to inflict a consistent and significant lesion to the hippocampus, resulting in hippocampally-dependent behavioral deficits and a long-lasting upregulation in neurogenesis, suggesting that this process might be a critical part of hippocampal recovery. In addition, we provide novel evidence of angiogenic and vasculature changes following hippocampal neuronal loss in CaM/Tet-DT_A_ mice. We posit that angiogenesis may be an important factor that promotes neurogenic upregulation following hippocampal neuronal loss, and both factors, angiogenesis and neurogenesis, can contribute to the adaptive response of the brain for behavioral recovery.

## Introduction

Neuronal loss is a common etiology of a variety of neurodegenerative disorders (Alzheimer's (AD), Parkinson's (PD), and Huntington's disease (HD) and brain traumas (stroke, epilepsy, and traumatic brain injury). Although pathologies and mechanisms underlying each of these disorders differ, including the affected brain regions, the common feature in all neurodegenerative disorders is the profound loss of neurons that results from the buildup of disease-specific protein aggregates and other cytotoxic downstream processes [1]–[3]. One of the greatest translational issues facing the field is overcoming the burden associated with neuronal loss, and effectively designing and evaluating novel therapies that can mitigate the loss of brain function due to neuronal cell death.

One brain region that is commonly affected in several neurological disorders and is critical in the learning and memory process is the hippocampus [4], [5]. Indeed, the hippocampus of patients with Alzheimer disease suffers from a loss of volume [1]–[3], which has been correlated with significant neuronal loss [6]. The hippocampus has also been shown to be susceptible to cell death following traumatic brain injury (TBI) [7], particularly during human development [8]–[11], and has also been observed in rodent models of TBI as well [12]. In addition, hippocampal sclerosis is also a frequently observed hallmark of temporal lobe epilepsy [13], [14]. Neuronal loss in the hippocampus following brain trauma or in neurodegenerative diseases has been linked to cognitive and memory deficits [15]–[23]. This evidence illustrates that the hippocampus is a critical brain area significantly affected in several neurodegenerative and brain trauma. Therefore, understanding the adaptive response of the hippocampus following neuronal loss may lead to novel therapies to alleviate these cognitive deficits.

Recovery of these deficits may be aided by the generation of new neurons in the hippocampus: one of the only brain regions capable of significant neurogenesis. Although once controversial [24]–[26], neurons can continue to differentiate in the adult brain from populations of neural stem cells in the subgranular zone (SGZ) in the hippocampus and the subventricular zone (SVZ) that lines the lateral ventricles [27]. Thousands of newborn cells can be generated every day [28], and although they demonstrate pruning and activity dependent survival [29], a portion can survive for several months or years in the adult human brain [30], [31]. The brains ability to generate new neurons presents a unique opportunity for recovery following hippocampal cell loss, however, the impact of this loss has on neurogenesis remains an understudied phenomenon.

To study the adaptive response of the hippocampus following neuronal loss, we used the innovative CaM/Tet-DT_A_ mouse model that induces hippocampal neuronal loss [32], [33]. This double transgene system consists of a transactivator driven by a constitutively active CaM-KII-alpha promoter, which in turns drive expression of a diphtheria toxin. Activation of diphtheria toxin expression is controlled by diet. Thus, this model provides a unique opportunity to study the adaptive response of the hippocampus following a selective neuronal loss, with a non-invasive method of lesioning.

In our studies, a 25-day lesion in the CaM/Tet-DT_A_ mice yielded significant neuronal loss in the CA1 and the dentate gyrus (DG) of the hippocampus but not in the entorhinal cortex (EC). Behavioral testing revealed significant performance deficits in a hippocampally-dependent Barnes maze task. In a second cohort of mice that had 3 months to recover post-lesion, training deficits in the Barnes maze persisted while long-term memory performance in a probe task recovered. Neurogenesis was also found to be upregulated in lesion mice compared to non-lesioned controls and this upregulation was long lasting. We also observed a correlation of neurogenesis upregulation with changes in angiogenesis. We conclude that neurogenic upregulation and angiogenesis following hippocampal neuronal loss may contribute to behavioral recovery.

## Materials and Methods

### Animal Use

3-month old CaM/Tet-DT_A_ mice were utilized in this study. All mice were housed with food and water *ad libitum* under a 12-hour dark/light cycle. All animal experimental procedures were performed in accordance with protocols approved by the Institutional Animal Care and Use Committee (IACUC) at the University of California, Irvine.

### CaM/Tet-DT_A_ Mouse Creation

Homozygous Tet-DT_A_ mice were bred with hemizygous CaM-tTA mice. All progeny received one copy of the Tet-DT_A_ gene, while half receive the CaM-tTA gene. Single and double transgenic mice were littermates, and only males were used. PCR was performed to confirm presence of the Tet-DT_A_ transgene using the primers 5′-TCTTCGTACCACGGGACTAA-3′ and 5′-CCGCAGCGTCGTATTTATTG-3′ and CaM-tTA using the primers 5′CGCATTAGAGCTGCTTAATG-3′ and 5′-TCGCGATGACTTAGTAAAGC-3′.

To provide a model of consistent and selective hippocampal neuronal loss, we employed the CaM/Tet-DT_A_ transgenic mouse [32], which was created by breeding TRE-DT_A_ mice [34] with CaMKIIα-tTA mice [35]. The transgene design is summarized in Figure S1, and briefly described here. The calcium-calmodulin dependent kinase II alpha (CaMKIIα) promoter drives expression of the transactivator (tTA) in the forebrain. In the absence of doxycycline, the tTA binds to the tetracycline responsive element (TRE), which in turn, drives expression of diphtheria toxin A chain (DT_A_). In the presence of doxycycline (supplied in the mouse diet), the tTA is sequestered, preventing binding to the TRE and expression of DT_A_. This model allows us to induce a lesion in the adult mouse and to control the length of induction.

**Figure 1:**
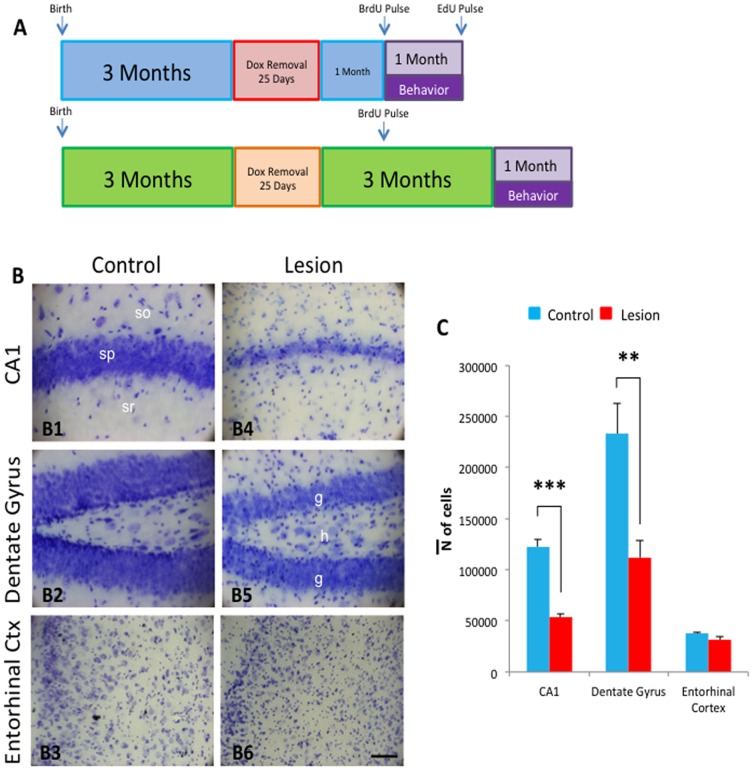
Experimental timeline and hippocampal cell loss following transgene induction in CaM/Tet-DT_A_ mice. **A**) Mice were aged for 3 months for normal development. Doxycycline was removed from the diet to induce a 25-day lesion to the CA1 of the hippocampus. The mice were given either 1 month or 3 months for recovery after traumatic lesion followed by hippocampal dependent behavioral tasks. One-week twice-daily pulse of BrdU through IP injection was given starting the 28^th^ day after dietary lesion and once-week once daily pulse of EdU through IP injection was given starting the 55^th^ day after dietary lesion to 1-month post lesion cohort. **B**) Light microscope images of Nissl staining of hippocampal CA1 (B1 and B4), dentate gyrus (DG) (B2 and B5), and entorhinal cortex (EC) (B3 and B6) subfields in control (B1–B3) and lesion (B4–B6) mice after 25 days of doxycycline removal and lesion induction. **C**) Stereological analysis in the pyramidal layer of the CA1 and granular layer of the DG reveal a significant reduction in the estimated total cell population in compared to control CaM/Tet-DT_A_ mice with no change in the EC. The values represent the mean±SEM (n = 5). **p<0.01, ***p<0.001. so: stratum oriens; sp: stratum piramidale; sr: stratum radiatum; g: granular layer; h: hilus. Scale bars: 100 µm.

Mice were maintained on doxycycline to prevent transgene expression *in utero* and during development. At 2 months and 4 months of age, doxycycline was removed from the diet for 25 days. On the 25^th^ day, doxycycline was returned to the mouse feed (with doxycycline water for 2 days to facilitate turning off the transgene). 2 months post-lesion, the mice were sacrificed. Their brains were fixed and sliced as described in the methods section. Serial slices were stained with Cresyl violet, and the hippocampal sections were imaged and analyzed using stereological optical fractionator. Representative images from the CA1 and dentate gyrus of control and lesion mice are presented in Figure 1, and total neuronal number was analyzed.

### DNA Isolation

DNA was isolated from tail samples for PCR analysis. Tails were digested in 480 µL STE plus 20 µL proteinase K (10 mg/mL) overnight at 55°C. Samples were centrifuged for 10 minutes at 13,200 rpm. Supernatant was then resuspended in 500 µL isopropanol. Precipitate was allowed to dry, then resuspended in 150 µL TE Buffer, and placed at 55°C overnight.

### Lesion Induction

Breeding pairs and weaned mice were maintained on doxycycline in food at a concentration of 2000 ppm (Research Diets Inc., New Brunswick, NJ). Food was replaced with regular food for 25 days to induce transgene expression. On the 25^th^ day, doxycycline food was returned to the diet, and water was replaced with 2 mg/mL doxycycline. Mice remained on doxycycline food until the completion of the experiment, regular water was returned after 3 days.

### Bromodeoxyuridine Labeling

To label maturing endogenous neuronal stem cells, mice were given a twice-daily intraperitoneal (IP) injection of bromodeoxyuridine at 50 mg/kg (BrdU, Sigma-Aldrich, St. Louis, MO), beginning on the 28^th^ day after doxycycline was returned to the diet, for five consecutive days (Fig. 1A).

### Ethynyldeoxyuridine Labeling

To label proliferating neuronal stem cells, the same cohort of mice were given once daily IP injection of Ethynyldeoxyuridine at 50 mg/kg (EdU, Invitrogen, Grand Island, NY), beginning on the 55^th^ day after doxycycline was returned to the diet for five days, and were sacrificed 24 hours after the last injection (Fig. 1A).

### Barnes Maze

Mice underwent a 5-day protocol of the Barnes Maze. The Barnes Maze consists of an elevated white platform with a diameter of 120 cm and 120 cm above the ground. At the edge of the maze were 40 holes of 5 cm diameter, equally spaced around the perimeter. Underneath one of the holes, an escape box was placed. The bottom of this box was covered with torn gauze bedding. Mice were trained for 4 days and underwent a probe trial on day 5. Before the first trial on the first day, mice were placed on the middle of platform and a box was placed over them. After 15 seconds, the box was removed and the mouse was allowed to explore the maze for a maximum of 120 seconds. If they found and entered the target chamber, they were returned back to their cage. If they did not find the target chamber within 120 seconds, they were led to the target. Mice underwent two trials a day with a 15-minute inter-trial interval. On the 5^th^ day, a 24-hour probe was performed, in which the target box was removed. Mice were again placed in the middle of the platform and allowed to explore for 120 seconds. Target head pokes (entries) and latency to find the target were measured.

### Tissue Preparation

Mice were deeply anesthetized with sodium pentobarbital and euthanized by perfusion transcardially with cold 0.1 M phosphate-buffered saline (PBS), pH 7.4. Half brain was fixed for 48 hours in 4% paraformaldehyde in 0.1M PBS (pH 7.4) and cryoprotected with 30% surcrose for immunohistochemical (IHC) analysis, whereas the other half was flash frozen in dry ice for biochemical analysis. Thick (40 µm) free-floating sections were obtained using a SM2010R freezing microtome (Leica Microsystems, Bannockburn, IL) and serially collected (each series contained sections that represented 1/6^th^ of the total brain) in cold PBS and 0.02% sodium azide solution, and stored at 4°C.

Protein extracts were prepared by homogenizing whole hippocampal hemisphere samples in 150 mg/mL of T-per extraction buffer (Pierce, Rockford, IL) cocktail, complemented with Complete Mini Protease inhibitor Tables (Roche, Indianapolis, IN) and 100 µL of 5 mM phosphatases inhibitors (Sigma-Aldrich, St. Louis, MO), followed by centrifugation at 100,000 g for 1 hour. Protein concentration in the supernatant was determined using the Bradford Assay.

### Immunocytochemistry and staining

Immunofluorecent or light-level immunohistochemistry followed standard protocols [36]. For fluorescent labeling, 4% paraformaldehyde fixed 40 µm thick free-floating sections were rinsed three times in PBS and then placed in blocking solution (PBS+5% normal goat serum+0.2% Tx-100) for one-hour. Sections were incubated with primary antibodies diluted in blocking solution overnight at 4°C. Following overnight incubation of primary antibody, sections were rinsed three times in PBS for 5 minutes each, sections were placed in appropriate secondary antibodies conjugated to Alexa 488, 555, or 635 fluorophores for one-hour (Molecular Probes, Grand Island, NY). Following one-hour incubation, sections were rinsed three times in PBS for 5 minutes each, mounted on glass slides, and cover-slipped using Fluoromount-G (Southern Biotech, Birmingham, AL). Light-level immunohistochemistry will follow a similar protocol except biotinylated secondary antibodies will be used and followed by incubation in avidin-biotin complex (ABC, Vector Labs, Burlingame, CA) and chromagen development in Diaminobenzidine. Primary antibodies utilized include NeuN (1∶1000, Millipore, Billerica, MA), S100B (1∶1000, abcam, Cambridge, UK), BrdU (1∶500, abcam, Cambridge, UK), EdU (1∶1000, Invitrogen, Grand Island, NY), DCX (1∶1000, abcam, Cambridge, UK), VEGF (1∶1000, abcam, Cambridge, UK).

### Cresyl Violet Stain

Sections were mounted on gelatin-coated slides and air dry for 24 hour in a dark room. Slides were re-hydrated in de-ionized H_2_O followed by PBS, pH 7.4, for 20 minutes each. Slides were then incubated in 1% Cresyl violet Acetate solution (Merck, refK28661940, Whitehouse Station, NJ) for 5 minutes following dehydration in graded ethanol's (70%, 96%+acetic acid, and 100%) and xylene for 5 minutes, respectively. Slides were cover-slipped using DPX (DBH) mounting medium (VWR, West Sussex, UK).

### Western Blot

Equal amounts of protein (10–20 µg, depending on protein of interest) of hippocampal homogenates were separated on 4–12% Bis-Tris Gel (Invitrogen, Carlsbad, CA), transferred to 0.45 µmol/L polyvinylidene difluoride membranes. Membranes were blocked for 1 hour in 5% BSA in 0.2% Tween-20 Tris-buffered saline (TBS-T) (pH 7.5). After blocking, the membranes were incubated overnight, at 4°C, with a primary antibody. The membranes were washed in TBS-T for 30 min and incubated at 20°C with the specific secondary antibody at a dilution of 1∶10000 (Pierce Biothechnology, Rockford, IL) for 60 minutes. The blots were developed using Super Signal (Pierce Biotechnology, Rockford, IL). Film were digitally scanned and analyzed by Image J software (NIH, Bethesda, MD) to measure signal intensity. Average signal intensity for each band was normalized to β -Actin bands, and then normalized to control. Primary antibodies utilized include VEGF (1∶1000, abcam, Cambridge, UK), GAPDH (1∶5000, Santa Cruz Biotechnology, Santa Cruz, CA), and β-Actin (1∶1000, Sigma-Aldrich, St. Louis, MO).

### Stereological Analysis of Cell Loss

All unbiased stereological assessments were performed using SteroInvestigator and Neurolucida softwares (MBF Bioscience, Williston, VT) [32], [37]. Briefly, an optical fractionator probe was utilized to estimate the total number of cells in the CA1 pyramidal cell layer and in the dentate gyrus (DG) granular cell layer. The stereological evaluation was performed on every 6^th^ section (40 µm coronal sections between 1.2 mm and 3.2 mm posterior to Bregma according to the atlas of Paxinos and Watson) of one brain hemisphere with n = 6 animals per group and per age. A counting frame of 40×40 µm in a sampling grid of 150×150 µm was used for the CA1, a counting frame of 50×50 µm in a sampling grid of 250×250 µm was utilized for the DG, and a counting frame of 40×40 µm in a sampling grid of 100×100 µm was used for the entorhinal cortex. Guard zone height for both top and bottom was set at 3 µm with an optical dissector height of 10 µm. The CA1 and dentate gyrus (DG) was defined using a 4× objective and the entorhinal cortex was defined using a 2.5× objective and the Nissl-stained cells were counted using a 100x/1.35 oil objective.

Cresyl violet-positive neurons were estimated using optical fractionator software from MBF Bioscience. Using the optical fractionator formula, in which N = 1/ssf.1/asf.1/hsf.∑Q, where ssf represents the section sampling fraction, asf is the are sampling fraction, which is calculated by dividing the area sampled with the total area of the layer, hsf stands for the height sampling fraction, which is calculated by dividing the height sampled (10 µm in this study) with the section thickness, and ∑Q is the total count of nuclei sampled for the entire area. The accuracy of the individual estimation was expressed by the total coefficient of error (CE) calculated using the CEs in each individual animal, with acceptable CE ranged between 0.02 and 0.07.

### Confocal Microscopy Analysis

Sections were imaged with a BioRad Radiance 2100 confocal microscope attached to an Olympus BX70 inverted microscope equipped with 488, 543, and 619 nm laser lines. All double and triple-labeled specimens will be imaged using the lambda-strobing function to prevent non-specific cross-excitation of fluorophores. For confocal Z-stack imaging, an appropriate confocal aperture will be selected using the automatic aperture feature. For comparative studies, identical laser and software settings will be utilized for all sections to be analyzed. Confocal microscopy analysis was performed on every 12^th^ section (40 µm coronal sections) of one brain hemisphere from 1.2 to 3.2 mm posterior of bregma with n = 12 animals per group. When analyzing BrdU labeled cells, slices were fluorescently stained for BrdU (488 nm), NeuN (555 nm), and S100B (635). When analyzing EdU labeled cells, slices were fluorescently stained for EdU (488 nm) and DCX (555 nm). Z stacks were taken using a confocal microscope at 40× magnification. Overlapping Z stacks were taken throughout the dentate gyrus, including the entire granular cell layer, subgranular zone, and hilus. Z stacks were stitched into one continuous 40× representation of the entire dentate for each slice using XuvStitch software (Xuvtools, Dreiländereck, Germany). For BrdU+/NeuN+ cells were considered new mature neurons, BrdU+/S100B+ cells were considered new mature astrocytes, and EdU+/DCX+ cells were considered new immature neurons.

### Statistical Analysis

All data are expressed as the mean ± SEM. All the quantitative data with multiple groups were analyzed using multifactor ANOVA with appropriate post-test (Dunnett's or Bonferroni's test). Comparisons between two groups (Control and Lesion) were performed by unpaired t-test. The acceptable level of significance for the tests was set at 95% confidence. All test were performed using Prism (GraphPad, La Jolla, CA).

## Results

### CA1 and dentate gyrus cell loss following transgene induction using unbiased stereology

Neuronal loss is a common component of various neurodegenerative diseases and brain injury, yet the adaptive response to the brain to recovery from this loss remains an understudied phenomenon. To address this, we engineered an inducible transgenic mouse that significantly ablates hippocampal neurons [32], [33]. Previous analysis from the lab revealed hippocampal cell loss by 20 days of induction, widespread cell death throughout the forebrain at 30 days induction, and induction beyond 30 days proved to be fatal. Additionally, previous work from the lab using a similar model, CaM/Tet-GFP, revealed significant loss in the CA1, CA3, DG, and EC by optical density [38]. However, stereological analysis is a more unbiased and quantitative method of analysis compared to optical density. For our experiments, we chose to utilize a 25-day lesion to ensure significant hippocampal cell loss and behavioral deficits in hippocampal-dependent tasks while sparing cortical function.

The stereological quantification revealed that after a 25 day lesion, CaM/Tet-DT_A_ mice have a significant decrease in the estimated total population of CA1 hippocampal pyramidal cells of 53,753±2,663 cells, meanwhile, control mice have 122,494±7,123 cells (*p<0.05, t-test), a decrease of 57% (Figure 1C). Similarly, stereological quantification of granular cell neurons in the dentate gyrus revealed a significant decrease of 50% (student's t-test, p<0.05) of the total population of the granular cells in CaM/Tet-DT_A_ lesion mice (111,976±16,174 cells, n = 5) versus control mice (223,013±30,206 cells, n = 5) (Figure 1C). Lastly, stereological quantifications of cortical neurons in the entorhinal cortex revealed no change (student's t-test, p<0.46) in total number of cells between CaM/Tet-DT_A_ groups (Lesion: 31,102±3847.5 cells, n = 5, Non-Lesion: 37,452.6±1677.5 cells, n = 5) (Figure 1C).

Additionally, we examined each of the 9 individual slices along the anterior/posterior axis of the CA1 and DG to investigate any regional differences in lesion severity (Figure S2). The quantification showed significant differences along the rostral to caudal axis (student's t-test, p<0.05) in both CA1 and DG.

### Recovery of Barnes maze performance in lesioned CaM/Tet-DT_A_ mice

**Figure 2:**
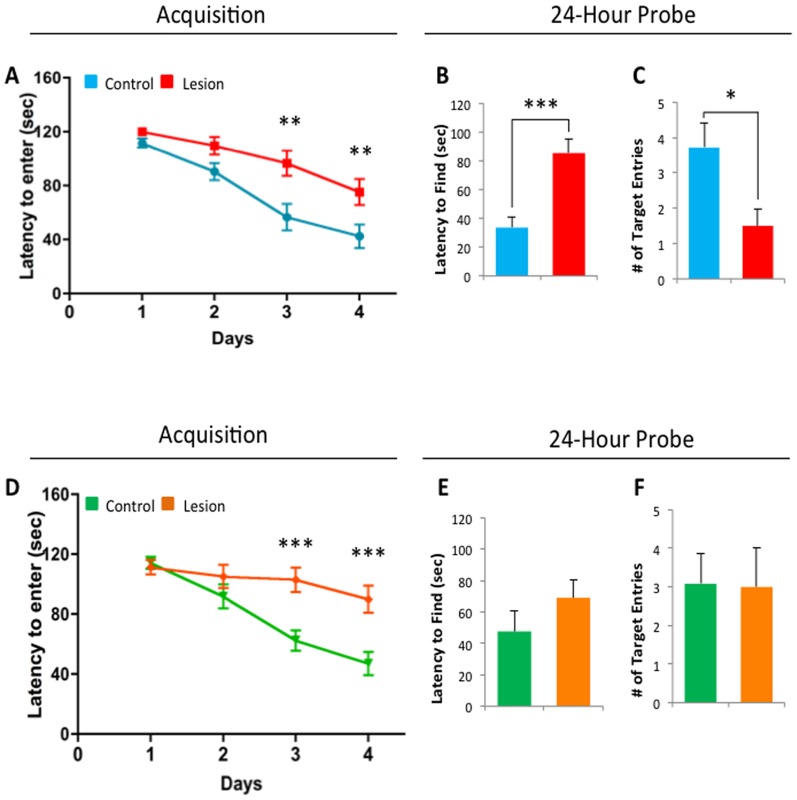
Recovery of 24-hour probe deficits, but not training deficits, 3 months post-lesion in the Barnes maze. **A**) 6-month old CaM/Tet-DT_A_ mice, lesion and control, were trained in the Barnes maze for 4 days. Significant learning deficits in the latency to enter the target on day 3 and 4 of training were observed in 1-month post lesion mice compared to control mice. **B**) In the 24-hour probe trial, 1-month post lesion mice had significantly higher latencies to find the target zone. **C**) Additionally, 1-month post lesion mice also exhibited significantly less correct head pokes into the target hole. **D**) CaM/Tet-DT_A_ lesion mice, exhibited significantly higher latencies to enter the target on day 3 and 4 of training compared to controls. **E**) 3-months post lesion mice exhibited no significant differences in the latency to find the target hole compared to controls. **F**) Additionally, 3-months post lesion mice revealed no difference in the total number of target entries. The values represent the mean±SEM (n = 12). *p<0.05, **p<0.01, ***p<0.001.

Next, we investigated the effects of hippocampal neuronal loss on behavioral performance, and investigated the possibility of behavioral recovery in the CaM/Tet-DT_A_ mouse model with time. To that end, both CaM/Tet-DT_A_ and control mice were tested at 1 month and 3 months after doxycycline removal (Figure 1A). We utilized the Barnes maze behavioral task, previously employed by our laboratory [39], which has been shown to assess spatial memory and hippocampal function [40]. Mice were subjected to 4 days of training in a Barnes maze, followed by a 24-hour probe test. During training, lesion mice exhibited significantly longer escape latency than control mice 1-month post lesion on days 3 and 4, as revealed by repeated measure ANOVA (Figure 2A). Similar deficits were also observed during training in the 3 months post lesion cohort (Figure 2D). 24 hours after the last day of training, a probe trial was performed, in which the target was removed and the mice were allowed to explore the arena for 120 seconds. The latency to first find the target hole was measured, as were the total number of entries (head pokes) into the target hole. 1 month post lesion, student's t-test revealed control mice exhibited significantly shorter latencies to find the target (control, time = 33.631±7.092 seconds, n = 14; lesion, time = 85.388±9.797 seconds, n = 12, p<.001), and significantly more correct entries (control, entries = 3.714±.683, n = 14, lesion, entries = 1.500±.469, n = 12, p<.05). 3 months post lesion, student's t-test revealed no significant difference in latencies to find the target (control, time = 47.658±13.325 seconds, n = 12; lesion, time = 69.075±11.366 seconds, n = 12, p<.05), and correct entries (control, entries = 3.083±.773, n = 12; lesion, entries = 3.000±1.015, n = 12, p<.05). To control for a possible difference in exploratory behavior and mobility, the total number of non-target entries were also observed, and yielded no difference between control and lesion mice by student's t-test 1 month (Figure S3A; control, entries = 15,357±1.659, n = 14, lesion, entries = 14.667±2.527, n = 12, p<.05) or 3 months post lesion (Figure S3B; control, entries = 27.667±3.581, n = 12; lesion, entries = 20.250±2.185, n = 12, p<.05).

### Neurogenesis is upregulated in the dentate gyrus of lesioned CaM/Tet-DT_A_ mice

Previous studies have indicated that cell death can have effects on neuronal proliferation in the hippocampus. Neurodegenerative disorders such as Alzheimer's disease [41], [42], Huntington's disease [43], [44] Parkinson's disease [45], [46], and epilepsy [47], [48] has been shown to alter neurogenesis. Neurogenesis has also been shown to be upregulated following brain injury, such as stroke [49], [50] and traumatic brain injury [51], [52]. However, many studies fail to investigate the duration of this neurogenic effect and whether neuronal survival or proliferation is primarily affected. Thus, we sought to provide a detailed look at possible neurogenic changes following hippocampal neuronal loss, and to provide a comprehensive examination of neurogenesis in the CaM/Tet-DT_A_ model and determine if neurogenesis might contribute to cognitive recovery observed in CaM/Tet-DT_A_ mice.

The timeline of our neurogenesis analysis is illustrated in Figure 1A. To measure neuronal survival, BrdU pulse was administered 1 month after the return of doxycycline to the diet (the end of lesioning in double transgenic CaM/Tet-DT_A_ mice), and was sacrificed 1 month after the BrdU pulse. At this point, proliferating neurons labeled with BrdU will mature, and express the mature neuronal marker NeuN [31]. Quantification of this population of BrdU+/NeuN+ cells is one of the most common and accurate ways to measure neurogenesis [53]. 1 out of every 12 hippocampal slices were analyzed, with more detail provided in the methods section. To quantify astrocytogenesis in the same samples, the astrocytic marker S100β was utilized, which labels the cell bodies of mature astrocytes [54]. Using confocal microscopy, the cellular proliferation marker BrdU, the mature neuronal marker NeuN, and the astrocytic marker S100β were analyzed in the dentate gyrus of lesioned and control mice. A BrdU+/NeuN+ cell was considered as an adult-born neuron, while a BrdU+/S100β+ positive cell was considered as an adult-born astrocyte.

**Figure 3:**
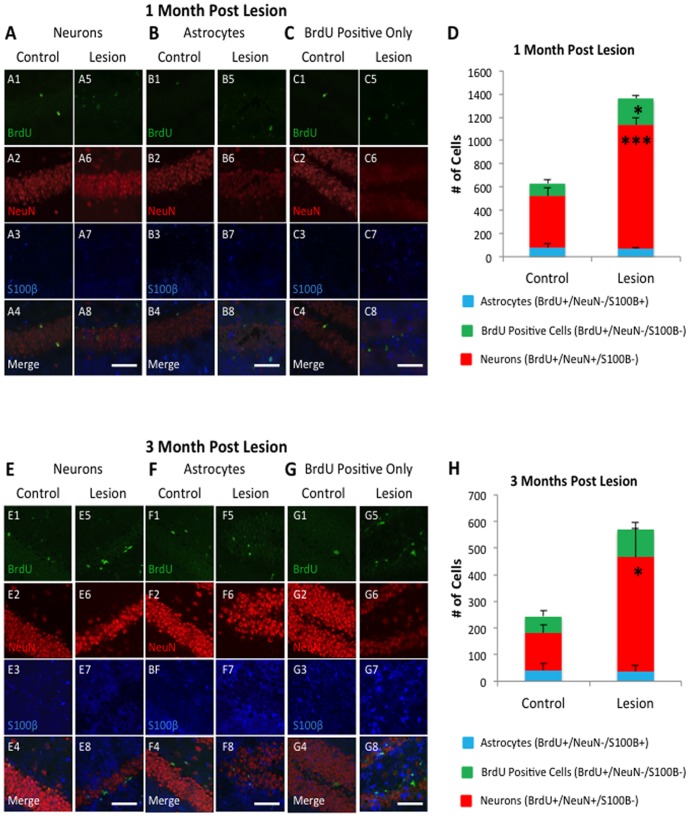
Neurogenesis is upregulated in CaM/Tet-DT_A_ mice 1 and 3 months post-lesion. Hippocampal slices from control (sub panels 1–4) and lesion (sub panels 5–8) mice were stained for the mature neuronal marker NeuN (sub panels 2 and 6), the astrocytic marker S100β (sub panels 3 and 7), and the proliferation marker BrdU (sub panels 1 and 5). The merged images are shown in sub panels 4 and 8. **D**) At 1 month post lesion, there was a significant increase in the number of BrdU+/NeuN+/S100β− cells in lesion mice, indicating an increase in neurogenesis. We also observed an increase in the number of BrdU+/NeuN/S100β− cells with an unclear differentiation. **H**) The upregulation of neurogenesis in lesion mice, as indicated by more BrdU+/NeuN+/S100β− cells, persisted for 3 months. Scale Bar: 50 µm.

Our cell counts revealed that neurogenic survival (BrdU+/NeuN+ cells) was significantly upregulated in lesion mice (1,068±64.287 cells, n = 5) compared to control mice (444±66.597 cells, n = 5) by student's t-test (p<.001), a 2.4 fold increase, illustrated in Figure 3A–D. No change in astrocytogenesis was observed (control, 79.2±32.110 cells, n = 5; lesion, 67.2±12.355 cells, n = 5, p<.05). In addition, we found a significant increase of BrdU+/NeuN−/S100β− cells in the hippocampus of lesion mice (223.2±30.735 cells) compared to control mice (108.0±31.061 cells) by student's t-test (p<.05). The majority of cells proliferating in the dentate differentiated into neurons (control 70.4%; lesion 78.6%), while only a subset became astrocytes (control 12.5%; lesion 4.9%), or another cellular type (control 17.1%; lesion 16.4%).

Though we have shown that hippocampal cell loss can cause a significant increase in neurogenesis, it remained unclear if these cells can survive for a long period of time. Neurons born during adulthood exhibit an activity-dependent survival [29], similar to that observed during development [55], suggesting the possibility that the previously observed upregulation could be short-lived. To measure long-term survival, we utilized a cohort of animals given a BrdU pulse 1 month post lesion, and were sacrificed 3 months later, allowing us to label and quantify adult-born cells that survive up to 3 months. In this stereologically-based analysis illustrated in Figure 3E–H, we still found that BrdU+/NeuN+/S100β− cells are significantly upregulated in lesion mice compared to controls (control 140.004±29.988 cells, n = 5; lesion 428.004±108.468 cells, n = 5, t-test, p<.05), a 3.0 fold increase. Overall, these data show that neurogenesis is upregulated after hippocampal neuronal loss, which may contribute to our observed behavioral recovery.

### Increased proliferation in the dentate gyrus following hippocampal lesioning

**Figure 4:**
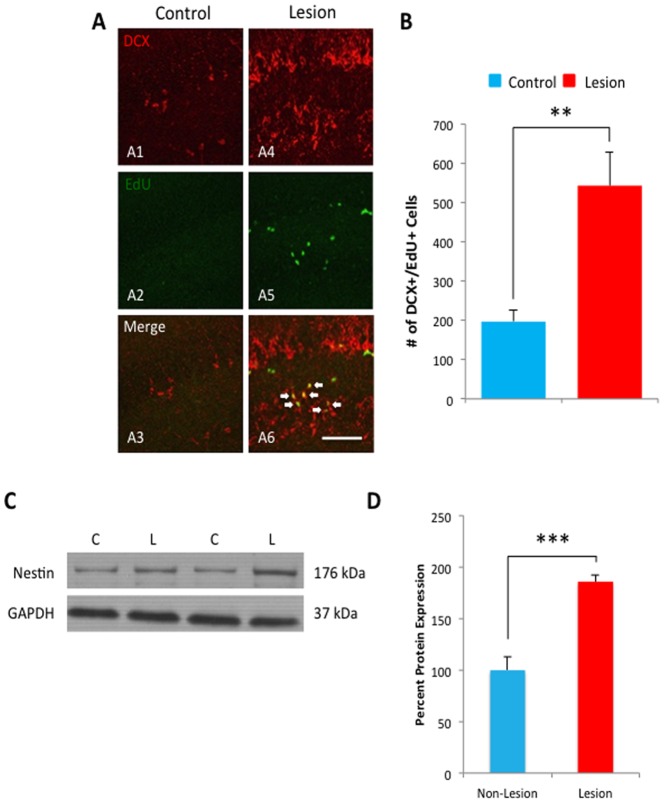
Upregulated proliferation of new-born neurons following lesion. **A**) Neuronal proliferation in the dentate of lesion (**A1–A3**) and non-lesioned (**A4–A6**) mice was analyzed in the 1 month post lesion group utilizing an alternate thymidine analoge, EdU. The EdU pulse was administered during the last 4 days before sacrifice, labeling newly dividing cells. The immature neuronal marker doublecortin (DCX) was used as a double label to confirm neuronal differentiation. **B**) Stereologically-based analysis revealed a significant increase in the number of EdU+/DCX+ cells in the dentate gyrus of lesioned mice compared to controls. **C**) Protein samples purified from lesioned and non-lesioned mice were analyzed by Western blot for levels of the Nestin protein, normalized to GAPDH, and quantified in **D**. The quantification showed a significant increase of Nestin in Lesion animals compared to control mice. Scale Bar: 50 µm.

We next sought to elucidate the effects of hippocampal cell loss on neuronal proliferation. To this end, we utilized a second thymidine analogue: EdU [56]. The EdU pulse was administered immediately before sacrifice, in the same cohort previously described, which will label newly proliferated cells. To specifically identify new neurons, we utilized the immature neuronal marker doublecortin (DCX). DCX is a microtubule-associated protein expressed in immature neurons during the first 3 weeks of maturation [57]. The dentate gyrus of 1 out of every 12 hippocampal slices was analyzed in lesioned and control mice. Fluorescent immunohistochemistry and confocal microscopy were utilized to count EdU+/DCX+ cells in the dentate, as illustrated in Figure 4A. Our data showed a significant increase in the number of EdU+/DCX+ cells in lesion mice compared to control mice (control 196.8±30.732 cells, n = 5; lesion 543±85.608 cells, n = 5, t-test, p<.01, Figure 4B), a 2.8 fold increase. In addition, western blot analysis showed that Nestin protein is significantly increased in lesion mice compared to control (n = 7, t-test, p<.006, Figure 4B). Thus, our findings indicate that neuronal proliferation is upregulated following hippocampal neuronal loss.

### Vasculature upregulation following hippocampal neuronal loss in lesioned CaM/Tet-DT_A_ mice

**Figure 5:**
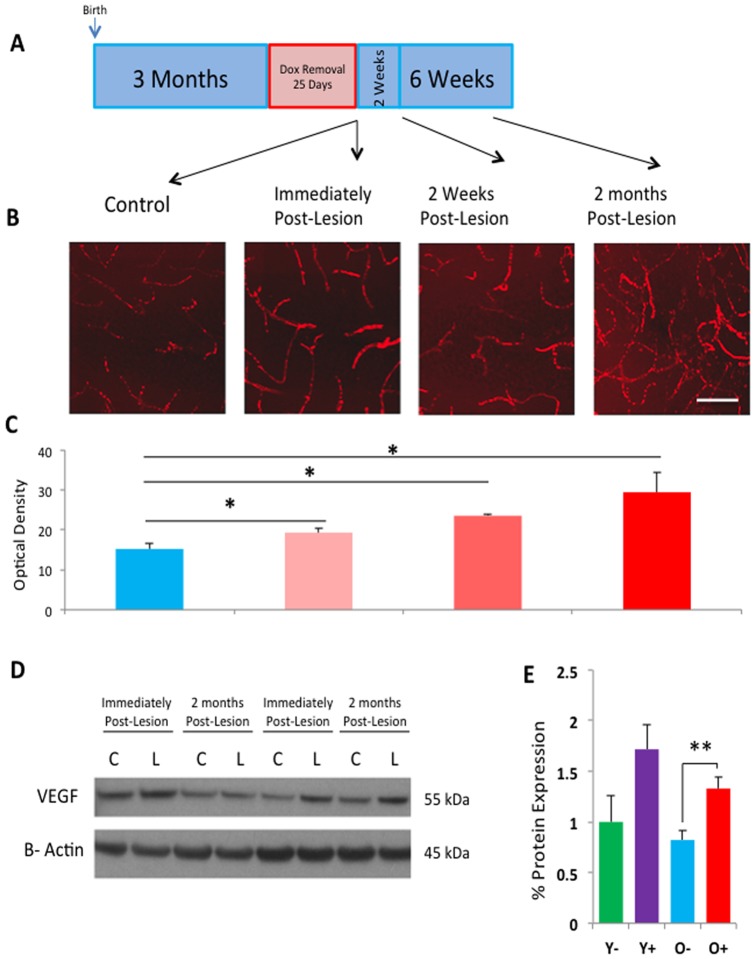
Increased angiogenesis and VEGF expression following lesion in CaM/Tet-DT_A_ mice. **A**) Angiogenesis was analyzed in control mice and lesion mice at 0, 2, and 8 weeks post lesion. **B**) Dextran-Texas red was administered during perfusion, labeling blood vessels in the dentate gyrus and imaged using confocal microscopy. **C**) Optical density was measured using Image J software, and revealed a step-wise increase in Dextran-Texas Red labeling following lesion. As VEGF has been shown to be a critical regulator of angiogenesis, **D**) VEGF expression was analyzed by Western blot using brain samples immediately post-lesion and 2 months post lesion. VEGF expression, normalized to actin, was increased in lesion mice compared to control mice, as illustrated in E. Scale Bar: 50 µm.

Next, we investigated the effects of hippocampal cell loss on angiogenesis. Changes in angiogenesis have been reported in previous models of brain injury, such as stroke [58]. Stroke-induced angiogenesis appears to be mediated by vascular endothelial growth factor (VEGF), which has also been implicated in trophic support for adult neurogenesis [59] and newly born neurons have been observed to migrate along vasculature following stroke [60]. To label angiogenesis, we utilized a fluorescently tagged Dextran-Texas Red compound that, when perfused into the bloodstream, labels the vasculature that can be imaged utilizing confocal microscopy [61]. We measured angiogenesis in four groups, (1) lesion and (2) non-lesion control mice sacrificed immediately on the 21^st^ day of doxycycline removal (lesion induction), (3) lesion mice sacrificed 2 weeks after the end of lesioning, and (4) lesion mice sacrificed 8 weeks after lesioning, the timeline of which is illustrated in Figure 5A. Upon sacrifice, mice were perfused with the Dextran-Texas Red compound. Confocal microscopy was used to image hippocampal slices, and z-stacks were performed in the dentate gyrus to visualize angiogenesis via the angiogenic marker Dextran-Texas Red, illustrated in Figure 5B. Mean optical density was measured using the image analysis software Image J. We found a step-wise increase in Dextran Texas-Red labeling, in which increasing lengths of time post-lesion resulted in significantly higher mean optical densities (0 week control, optical density = 15.24, n = 4; 0 week lesion, optical density = 19.34, n = 4; 2 week lesion, optical density = 23.54, n = 4; 8 week lesion, optical density = 29.46, n = 4, one-way ANOVA, Figure 5C).

We next sought to biochemically investigate possible factors that could contribute to this angiogenic upregulation, in particular, vascular endothelial growth factor (VEGF). VEGF has previously been shown to promote angiogenesis [62] and elevated levels of VEGF have been reported following brain injury [59]. The steady state levels of VEGF was measured by Western blot, and the data showed a significant increase in VEGF expression in CaM/Tet-DT_A_ mice post-lesion compared to non-lesion controls (n = 5, t-test, p<.01, Figure 5D and 5E). Taken together, these data indicate that angiogenesis is upregulated after a severe brain damage and it might provide trophic support for neurogenic upregulation.

## Discussion

In the current study, we had demonstrated significant and selective cell loss in the hippocampus (57% in the CA1) of an inducible CaM/Tet-DT_A_ mouse model, which results in specific hippocampal-dependent behavioral deficits one month following lesioning. Learning deficits persist even 3 months post lesion, as evidenced by higher latencies in Barnes maze training. Performance deficits in the 24 hour probe trial were evident shortly (1 month) after lesioning compared to controls, however, they were ameliorated 3 months post lesion. Previous studies have reported deficits in spatial learning following hippocampal lesion [22], [63], and the extent of deficits can be determined by the extent of lesioning [64] or even sub-regional differences [65]. In our study, the newborn neurons produced in response to lesion may be selectively recruited for long-term memory retrieval rather than acquisition, possibly accounting for the partial behavioral recovery observed. While previous studies investigating the role of neurogenesis in learning and memory have produced mixed results [66]–[68], there have been observed reports in which neurogenesis significantly and preferentially improves performance in long-term spatial memory [69], [70], although strong evidence points to a specific role of neurogenesis and the dentate gyrus in pattern separation tasks [71]–[73]. However, future experiments utilizing time-specific inactivation of adult-born neurons during acquisition or probe trials would provide more direct evidence to determine the specific role of neurogenesis in these tasks.

Alterations in neurogenesis have been previously observed in a variety of neurodegenerative disorders, such as Alzheimers [41], [42], Huntington's [44], and Parkinson's disease [74]. However, it is unclear if neurogenesis is a compensatory mechanism that contributes to recovery, or if changes in neurogenesis can somehow contribute to disease progression. In general, increases in neurogenesis have been correlated with improvements in cognition [66], [68], while deficits in neurogenesis are associated with cognitive decline [75], [76]. Other studies directly inhibiting neurogenesis in animal models have indicated adult-born neurons in the hippocampus play a significant role in hippocampal function [67], [77]–[80]. In cases of hippocampal neuronal loss, in particularly in our CaM/Tet-DT_A_ model, upregulated neurogenesis may alleviate cognitive deficits by cell replacement, or by contributing trophic factors to the post-injury environment. In addition to an upregulation in neurogenesis, we also observed an increase in cell proliferation that was not neuronal or astrocytic. These cells are likely composed of oligodendrocytes or microglia [81]. Microglia have been shown to activate and migrate to sites of brain injury [82], [83] and can proliferate from microglia progenitor cells [84].

We also observed an increase in vascularization in the dentate gyrus of lesioned mice, and an increase in the levels of vascular endothelial growth factor (VEGF) protein in the forebrain. Angiogenic changes following brain injury are not unprecedented, and have been shown to be upregulated following stroke [58], and mediated by VEGF. VEGF can promote angiogenesis by binding to specific cell surface receptors [85]. Interestingly, VEGF has been shown to directly upregulate neurogenesis as well [59], [86]. As newborn neurons migrate along vasculature in the rostral migratory stream [87], and VEGF mediates this process [88], the angiogenic changes we observe in the hippocampus of CaM/Tet-DT_A_ mice may help alter the neurogenic niche to promote proliferation of newborn neurons. However, future studies employing anti-angiogenic compounds would help determine if angiogenesis can directly upregulate neurogenesis in our model.

Taken together, our evidence clearly illustrates that the post-injury environment has a host of factors that can be affected, such as neurogenesis and angiogenesis, and that focusing on any one factor may not paint a clear picture of the mechanisms at work during recovery following neuronal loss. The robust and long-lasting effect on neurogenic upregulation suggests that this may help play an important role in recovery. Indeed, inducing neurogenesis pharmacologically has been explored as a therapeutic approach following brain trauma following TBI [89], [90]. However, it is worth nothing that in the case of neurogenesis, more is not always better. Aberrant and upregulated neurogenesis has been implicated as a possible factor in the disease pathogenesis of epilepsy [47], [91], and neurogenesis may actually contribute to forgetfulness in certain circumstances by competing with existing hippocampal networks [92]. Our in-depth characterization of neurogenesis in the CaM/Tet-DT_A_ mouse now provides us with a starting point to better understand how these adult-born cells are affected by neuronal loss, and how they may be utilized to aid in brain repair. Further studies utilizing this model may shed more light on the mechanisms of neurogenesis upregulation, and identify novel therapies for boosting recovery following neuronal loss.

## Supporting Information

Figure S1 **CaM/Tet-DT_A_ mouse model of selective neuronal ablation.** The calmodulin-dependent kinase II alpha (CaMKIIα) drives expression of the transactivator (tTA) in the forebrain. In the absence of doxycycline, the tTA binds to the tetracycline responsive element (TRE), which in turn, drives expression of diphtheria toxin A chain (DT_A_). In the presence of doxycycline (supplied in the mouse diet), the tTA is sequestered, preventing binding to the TRE and expression of DT_A_ (adapted from Yamasaki et al, 2007).
(TIF)

Figure S2 **Rostral-caudal axis cell loss in CA1 and DG in CaM/Tet-DT_A_ mice following 25 days of transgene induction.** The stereological data from Figure 1 was broken down to examine slice by slice differences along the anterior/posterior axis in the hippocampus. **A**) Cell loss in the CA1 was most pronounced in the most anterior slices, and notably absent from the most posterior slices analyzed. **B**) Cell loss was more homogenous throughout the dentate gyrus, though is spared in the most anterior portion of the hippocampus.
(TIF)

Figure S3 **No changes in Barnes target exploration performance between lesion and control mice.** During Barnes maze analysis, the total number of non-target entries was measured as a control for total exploration. There were no significant differences between control and lesion mice (**A**) 1 month post lesion or (**B**) 3 months post lesion.
(TIF)
