# Supplementary figures and images for: Hippocampal Adaptive Response Following Extensive Neuronal Loss in an Inducible Transgenic Mouse Model
Source: PLoS One. 2014 Sep 3;9(9):e106009. doi: 10.1371/journal.pone.0106009 (PMC4153578; doi:10.1371/journal.pone.0106009)

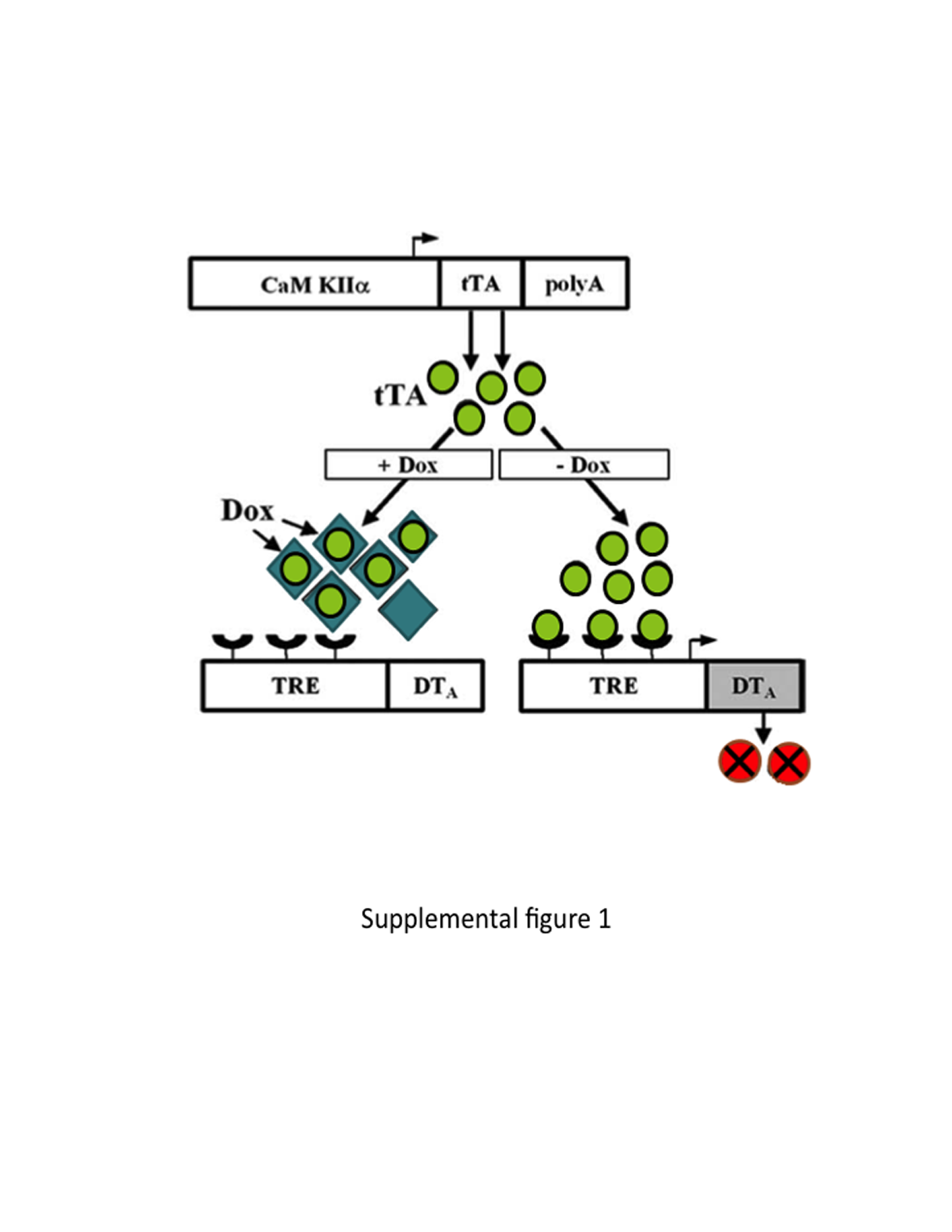

Supplement: Figure S1 — CaM/Tet-DTA mouse model of selective neuronal ablation. The calmodulin-dependent kinase II alpha (CaMKIIα) drives expression of the transactivator (tTA) in the forebrain. In the absence of doxycycline, the tTA binds to the tetracycline responsive element (TRE), which in turn, drives expression of diphtheria toxin A chain (DTA). In the presence of doxycycline (supplied in the mouse diet), the tTA is sequestered, preventing binding to the TRE and expression of DTA (adapted from Yamasaki et al, 2007). (TIF) [file pone.0106009.s001.tif]

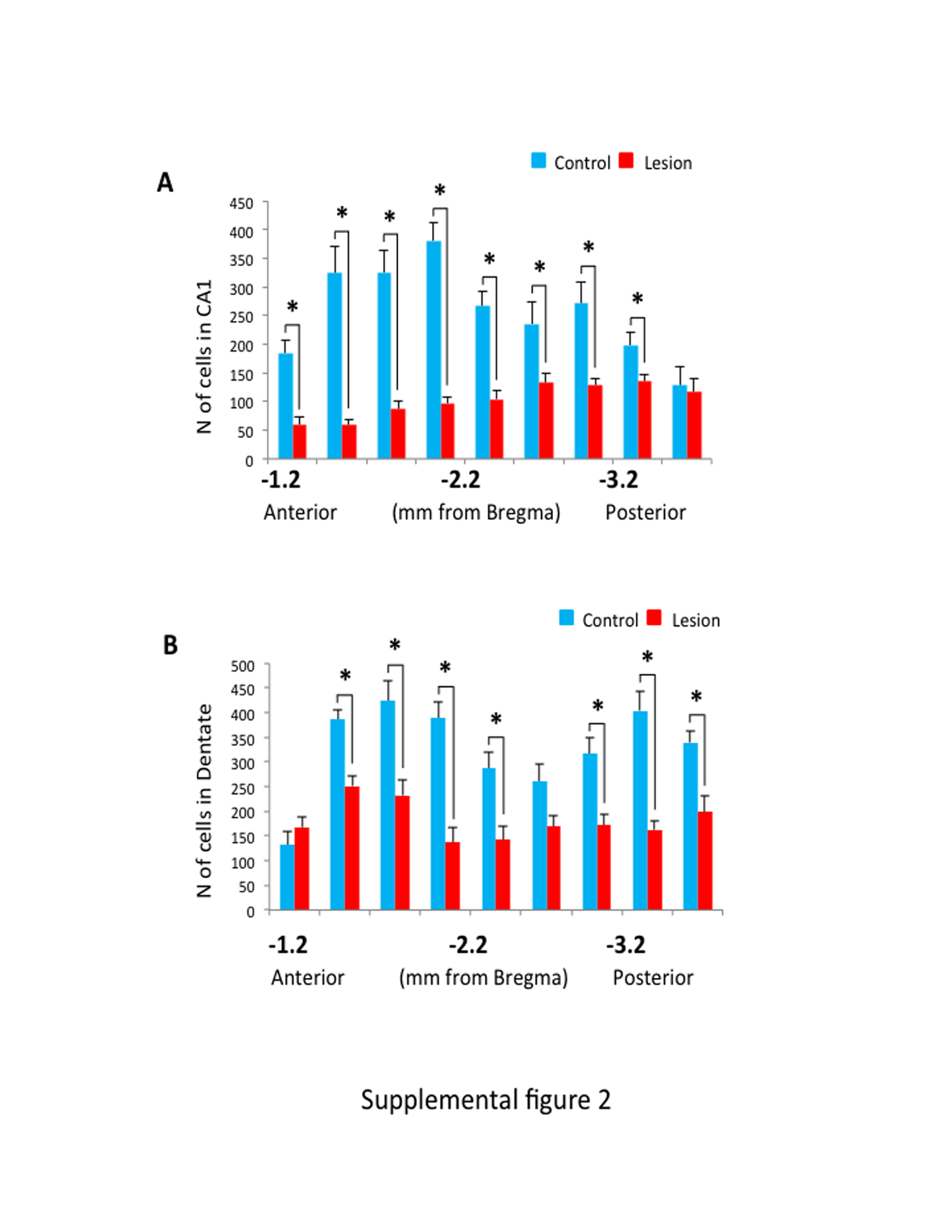

Supplement: Figure S2 — Rostral-caudal axis cell loss in CA1 and DG in CaM/Tet-DTA mice following 25 days of transgene induction. The stereological data from Figure 1 was broken down to examine slice by slice differences along the anterior/posterior axis in the hippocampus. A) Cell loss in the CA1 was most pronounced in the most anterior slices, and notably absent from the most posterior slices analyzed. B) Cell loss was more homogenous throughout the dentate gyrus, though is spared in the most anterior portion of the hippocampus. (TIF) [file pone.0106009.s002.tif]

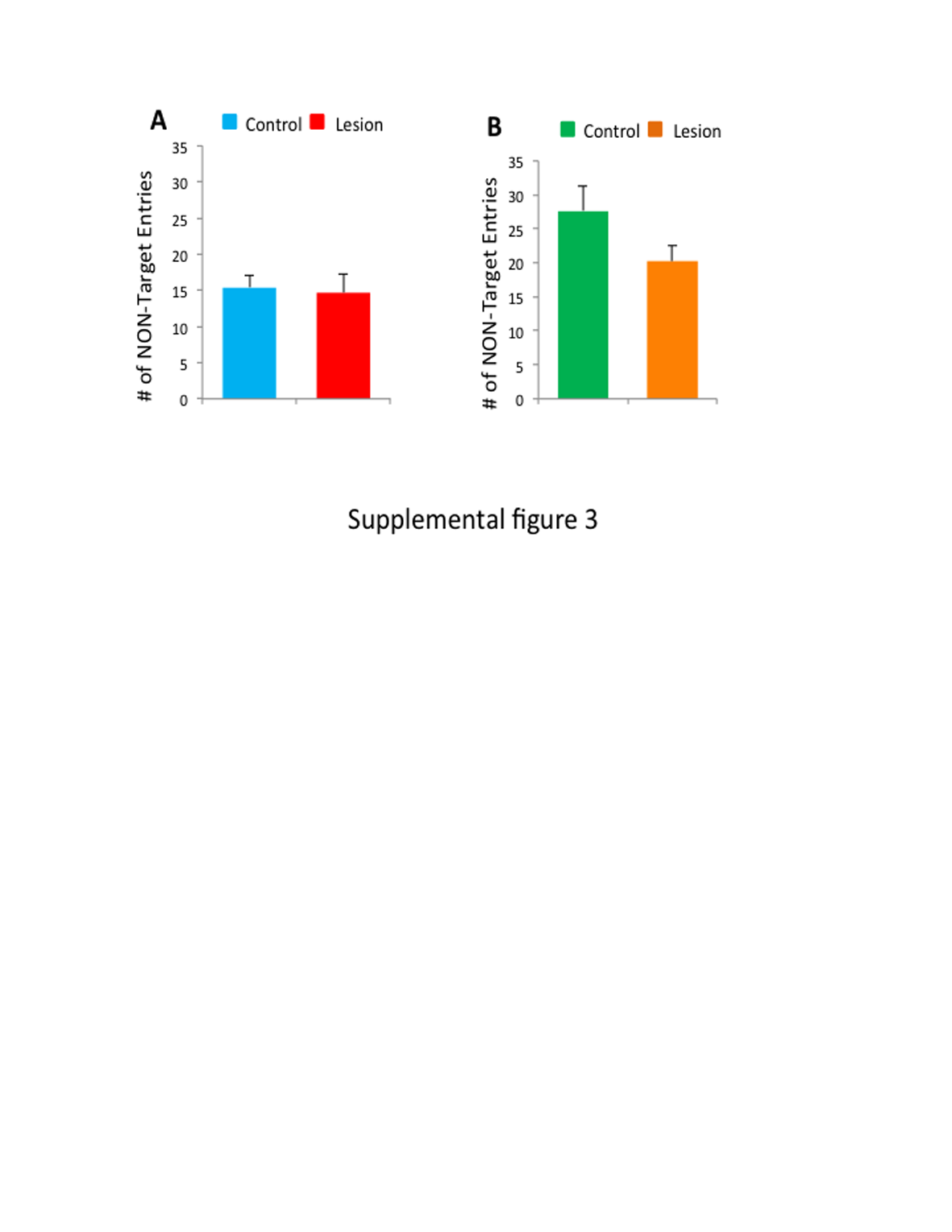

Supplement: Figure S3 — No changes in Barnes target exploration performance between lesion and control mice. During Barnes maze analysis, the total number of non-target entries was measured as a control for total exploration. There were no significant differences between control and lesion mice (A) 1 month post lesion or (B) 3 months post lesion. (TIF) [file pone.0106009.s003.tif]
